# Impacts of Water Level on Multidimensional Waterbird Diversity in a Wetland of Yangtze River Floodplain: Insights for Conservation

**DOI:** 10.3390/ani16213354

**Published:** 2026-10-24

**Authors:** Shengxin Liu, Lujing Zhou, Haoxue Gong, Qiang Wu, Yong Zhang

**Affiliations:** Co-Innovation Center for Sustainable Forestry in Southern China, College of Life Sciences, Nanjing Forestry University, Nanjing 210037, China; 2073126457@njfu.edu.cn (S.L.); zhou990114@163.com (L.Z.); 15564468411@163.com (H.G.); qiangwu@njfu.edu.cn (Q.W.)

**Keywords:** hydrological regime, waterbird diversity, effect pathways, temporal variation, conservation practices

## Abstract

Anthropogenic hydrological modifications have profoundly altered natural hydrological regimes, which are widely acknowledged as a key driver of waterbird diversity in wetland systems. However, the pathways linking water level to multidimensional waterbird diversity and seasonal variation remain poorly understood, limiting targeted conservation and sustainable wetland management. To address these, based on an annual survey data from Shijiu lake in the Yangtze River Floodplain, we examined the direct and indirect effects of water level on the taxonomic, functional, phylogenetic diversity of waterbirds across annual, wintering, and non-wintering periods. Piecewise structural equation models showed that water level influenced multidimensional waterbird diversity primarily through indirect pathways mediated by local habitat conditions, including water area (WA) and vegetation condition (NDVI). The strength of these indirect pathways differed between the wintering and non-wintering periods. Moreover, generalized additive models revealed seasonally distinct response patterns of multidimensional waterbird diversity to these habitat variables, consistent with the seasonally mediated effects of water level. Overall, our results reveal seasonal differences in waterbird community responses to water level changes. These findings underscore the need for targeted, seasonally responsive hydrological management following future dam construction in lakes and provide practical guidance for waterbird conservation in Shijiu Lake and similar wetland ecosystems.

## Simple Summary

The hydrological regime of lake wetlands shapes landscape structure and creates diverse habitats for waterbirds. Although altered hydrological regimes are known to change waterbird community composition, how water level influences waterbird diversity remains poorly understood. In this study, we investigated Shijiu Lake, a lake wetland in the lower Yangtze River, using 12 months of waterbird surveys, water level data, and remote sensing data to examine the effects of water level on three dimensions of waterbird diversity—taxonomic, functional, and phylogenetic diversity—across different periods. Piecewise structural equation models showed that water level fluctuations mainly influence multidimensional waterbird diversity indirectly by reshaping local habitat conditions, rather than directly, and that these effects vary by season. These findings suggest that, following future dam construction in lakes, water level management should be seasonally adjusted to protect waterbird diversity while balancing conservation with social and economic development.

## 1. Introduction

Wetland ecosystems are among the most important ecosystems on Earth, playing a crucial role in maintaining biodiversity, regulating regional hydrological cycles, and providing protection from drought [1,2]. As a major wetland type, lake wetlands are not only essential for human well-being, but also provide ideal habitats for numerous organisms [3,4]. Among them, waterbirds are a key ecological group which depends on diverse habitats created by periodic hydrological fluctuations to complete their life cycles in wetlands [5]. So far, although wetland loss has been recognized as one of the major drivers of waterbird diversity declining [6], loss of habitat functions driven by hydrological changes are often subtle since the wetland extent remains unchanged [7]. This kind of hidden loss has been received some attention [8], yet the effect pathways of hydrological regimes on waterbird diversity remains largely understudied, hindering targeted conservation practices and sustainable development.

In wetlands, the hydrological regime acts as the primary driving force in determining landscape patterns and configurations, vegetation development, and consequently waterbird diversity [9,10]. Under natural conditions, water levels fluctuate seasonally with precipitation and runoff, creating the dynamic habitat conditions on which waterbird communities depend. In the Anthropocene, however, intensifying human activities have substantially altered these regimes. Over the past century, a large number of dams have been constructed globally [11], dramatically modifying water volume, water level fluctuations and hydrological connectivity. Although these dams provide important services, they have simultaneously induced cascading effects on the habitat quality and quantity for waterbirds [12,13]. In contrast to natural fluctuations, dam regulation superimposes managed water level fluctuations on natural climatic rhythms, producing regimes that differ fundamentally from natural fluctuations. These alterations highlight the need to understand how natural water level dynamics influence waterbird diversity, providing a natural baseline for waterbird conservation in the face of ongoing hydrological alteration, including dam construction. However, the mechanisms underlying this relationship remain poorly understood, especially regarding the relative contributions of direct effects and indirect pathways mediated by changes in habitat conditions.

In addition, waterbirds utilize wetlands in different ways across seasons, reflecting seasonal differences in ecological requirements. Furthermore, habitat requirements of the same species may also differ across their life history stages [14]. For example, during the breeding period, waterbirds require suitable habitat for nesting and reproduction, whereas during the wintering period, their habitat use is more strongly associated with suitable foraging and roosting habitat. These seasonal differences in habitat use and ecological requirements may contribute to seasonal variation in waterbird diversity responses to hydrological regimes [15]. Recent studies in Yangtze River floodplain lakes have demonstrated that altered water level regimes can reduce suitable habitats and modify the functional composition of waterbird assemblages, particularly under prolonged high water levels during winter. These findings highlight the importance of maintaining seasonal hydrological fluctuations and heterogeneous shallow water and mudflat habitats to support waterbird conservation [16,17]. Therefore, understanding the mechanisms by which water level influences waterbird diversity across different temporal scales is a priority for informing effective conservation strategies.

The Yangtze River Basin harbors one of the most extensive river-lake-wetland network in the world, supporting the livelihoods of approximately one-third of China’s population [18,19]. Meanwhile, the Yangtze wetlands, functioning as one of the most important wintering areas in the East Asian-Australasian Flyway, support more than one million waterbirds every winter [20]. To date, more than 50,000 dams have been constructed in the Basin over the past century [21]. Consequently, hydrological regimes in wetlands have been changed and thereafter affect waterbird communities [22,23]. Shijiu Lake, an important wetland in the lower Yangtze River floodplain, serves as a major wintering ground for migratory waterbirds from October to March, including the Tundra Bean Goose (*Anser fabalis*), Tundra Swan (*Cygnus columbianus*), Eurasian Teal *(Anas crecca*), Dunlin (*Calidris alpina*), Oriental Stork (*Ciconia boyciana*), and Eurasian Spoonbill (*Platalea leucorodia*). From June to September, the lake also provides breeding habitat for species such as the Whiskered Tern (*Chlidonias hybrida*), Grey-headed Lapwing (*Vanellus cinereus*), Pheasant-tailed Jacana (*Hydrophasianus chirurgus*), Cotton Pygmy Goose (*Nettapus coromandelianus*), and Great Crested Grebe (*Podiceps cristatus*) [24]. As one of the three lakes naturally connected to the lower Yangtze River, Shijiu Lake is an ideal site for examining how water level fluctuations affect waterbird diversity across different temporal scales.

In this study, we used monthly waterbird survey data (November 2024–October 2025) from Shijiu Lake, a human-impacted wetland ecosystem undergoing ecological restoration, to assess the direct and indirect effects of water level fluctuations on waterbird diversity at three temporal scales: year-round, wintering (October 2024–March 2025), and non-wintering (April–September 2025) period. We quantified taxonomic diversity using the Shannon–Wiener index, and standardized functional and phylogenetic diversity to examine the direct and indirect effects of water level fluctuations on waterbird diversity across temporal scales. We tested the following two hypotheses: (1) water level, as a key driver of waterbird habitat conditions, governs multidimensional waterbird diversity mainly through indirect effects across different temporal scales; (2) moderately elevated water level may enhance multidimension waterbird diversity during the wintering period, when water levels are typically low. In contracted reduced water levels during the non-wintering period, when water levels are generally higher, would promote multidimensional diversity.

## 2. Materials and Methods

### 2.1. Study Area

**Figure 1:**
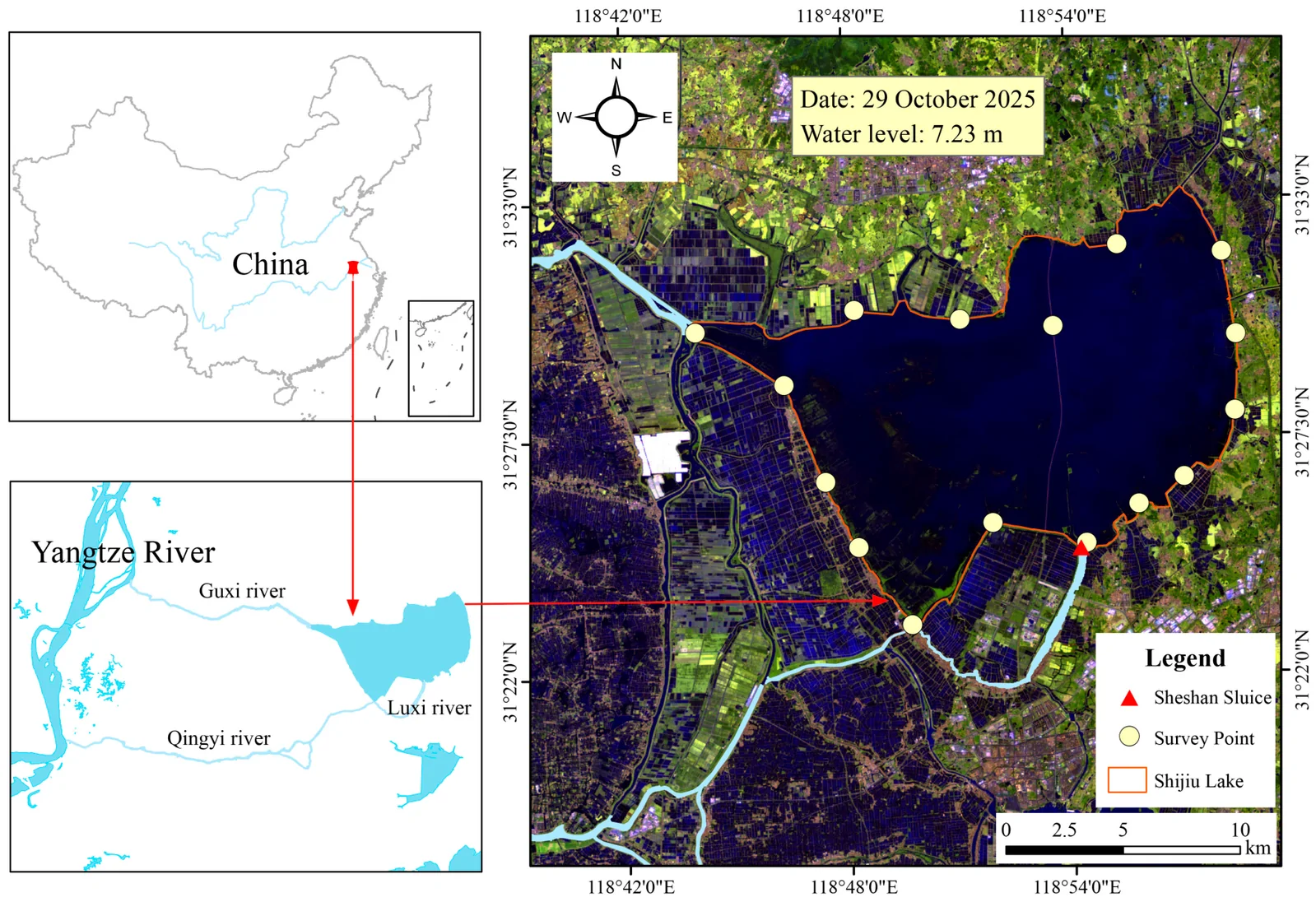
Location of Shijiu Lake in the lower Yangtze River, China and the layout of waterbird survey sites in this study. On the right side of the figure, the green area represents emergent vegetation and the blue area represents water.

Shijiu Lake (31°23′–31°33′ N, 118°43′–118°58′ E), covering a total area of 214 km^2^, is located at the junction of southwestern Jiangsu and eastern Anhui provinces, the lake basin is irregularly quadrangular (Figure 1). Shijiu Lake shares a subtropical humid monsoon climate, with a mean annual temperature of 15.7 °C and an average annual precipitation of 1100 mm. The lake directly connects to the Yangtze River via the Guxi and Qingyi river, and the lake water level seasonally fluctuates normally in tandem with changes in the Yangtze River water level. Seasonal water level fluctuations create distinct habitats: summer inundation nourishes plenty of aquatic vegetations (*Phragmites australis*, *Typha orientalis*, *Trapa bispinosa*), offering an ideal place for waterbirds to breed, whereas winter drawdown exposes mudflats and Carex meadows which provide key foraging grounds for wintering waterbirds. Sheshan Sluice serves as a pure flood spillway linking the lake to the Yangtze River via the Luxi and Qingyi river.

### 2.2. Water Level Data

**Figure 2:**
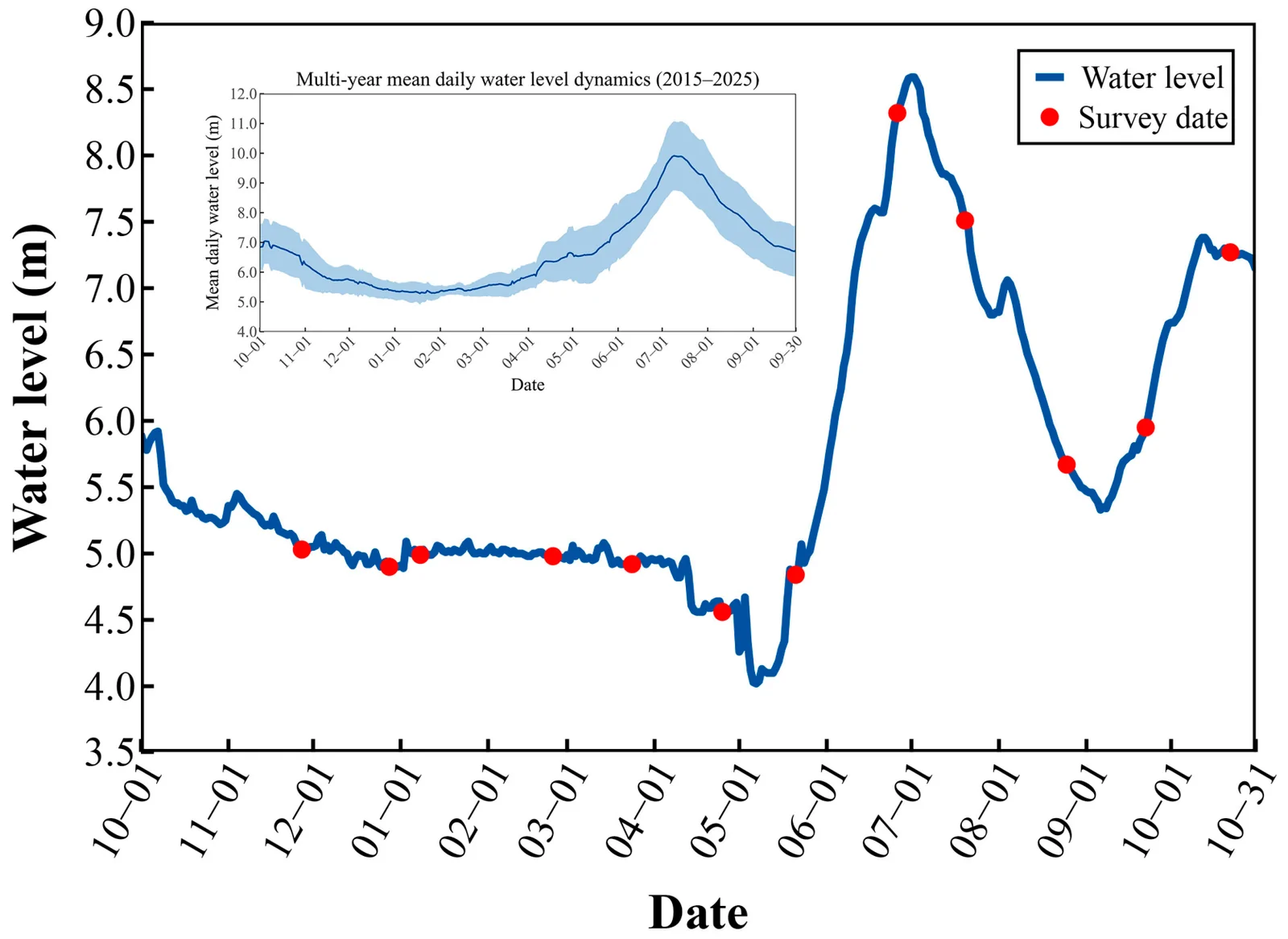
Daily water level trends and monthly waterbirds survey dates at Shijiu Lake from 1 October 2024 to 31 October 2025. The embedded figure displays the daily water level trend of Shijiu Lake from 2015 to 2025 and the blue shaded area represents 95% confident intervals.

We obtained daily water level data (Wusong Elevation) from the Jiangsu Provincial Hydrological Information Network (https://jswater.jiangsu.gov.cn/). These data represent daily observations recorded at the Sheshan Sluice at 8:00 a.m, Beijing time between 1 October 2024, and 31 October 2025 (Figure 2). During the study period, although Shijiu Lake experienced relatively low water levels compared to previous years over the past decade, the overall trend of fluctuations was analogous (Supplementary Figure S1).

### 2.3. Climate Data

The daily mean temperature and daily total precipitation data were obtained from the Lishui district meteorological station, the nearest meteorological station to Shijiu Lake, via the China Meteorological Data Service Center (CMDC, https://data.cma.cn/, 28 November 2025). We subsequently computed the monthly coefficient of variation (CV) for temperature and precipitation to characterize their intra-month variability.

### 2.4. Waterbird Surveys

We established 16 waterbird monitoring sites covering the whole of Shijiu Lake (Figure 1). Survey sites were chosen based on the topography, geomorphology, and seasonal water level fluctuating patterns of the lake. Surveys were conducted monthly from November 2024 to October 2025, resulting in a total of 12 survey rounds. Field surveys were carried out on days with good visibility (i.e., clear weather, no strong wind, and no fog), between 07:30 and 17:00. Waterbird species and abundances were recorded using binoculars (Shuntu Kunming, China, 8 × 40) and telescopes (Swarovski Optik, Absam, Austria, 20–60×). During each survey, waterbirds at each site were identified and counted, with the process completed in approximately 5–15 min per site. Each monitoring site encompassed a circular area with a 1.5 km radius (measured by rangefinder). To avoid double counting at individual survey sites, observers were divided into two survey teams that simultaneously surveyed different sites while moving in opposite directions toward each other along the lake, meeting midway and completing all sites within a single day. Each team was led by an observer with at least three years of waterbird monitoring experience, and carried out surveys throughout the study period to ensure consistency in species identification and counting [25]. Only waterbirds flying over the area were ignored.

### 2.5. Habitat Data

We downloaded the Sentinel-2 and Landsat imagery data from the European Space Agency (https://dataspace.copernicus.eu, 19 November 2025) and the USGS (https://www.usgs.gov, 20 September 2025), respectively (for detail information see Supplementary Table S1). First, we defined the study area of Shijiu Lake using the water extent extracted from the satellite image acquired on 4 August 2024 (water level: 10.11 m), representing high water conditions, cloud-free imagery, and proximity to the start of the study. To reduce uncertainty, we selected 12 satellite images (with cloud cover < 10%) which were temporally close to the survey dates. Because of the absence of high-quality images in June and July 2025, we used images acquired on 3 July and 17 June 2025 as substitutes, respectively. This threshold ensured consistency between water levels at the time of image acquisition and field surveys [26]. Prior to image interpretation, Sentinel-2 data were resampled to a spatial resolution of 30 m. Second, we classified land cover within the lake area into four categories—open water, mudflat, grassland, and emergent vegetation—using the normalized difference vegetation index (NDVI) and the modified normalized difference water index (MNDWI) [27,28]. Finally, land cover proportions within a 1.5 km radius of each waterbird survey site were extracted according to the spatial extent of the waterbird survey at each site to characterize local habitat conditions [29]. Landscape diversity metrics, including Simpson’s diversity index (SIDI) and Shannon’s evenness index (SHEI), were calculated for subsequent analyses. The buffer size was selected to represent the typical spatial scale of waterbird habitat. Remote sensing image processing was conducted using ENVI 5.6 and ArcMap 10.8.1, and landscape metrics were calculated using Fragstats 4.2.

### 2.6. Diversity Indices Calculation

We calculated monthly waterbird diversity across three dimensions for each monitoring site: taxonomic (Shannon–Wiener index, H), functional (Standard effect size of functional diversity, SES.FD), and phylogenetic (Standard effect size of phylogenetic diversity, SES.PD), the specific formulas were as follows:

$$H\;=\;-\sum_{i=1}^{S}P_{i}\ln P_{i}$$

where S is the total number of species observed, and Pi represents the individual number proportion of the ith waterbird species within a community [30].

*SES* = (*M_obs_* − *M_null_*)/*SD_null_*

where M_obs_ is the observed value of FD or PD, M_null_ is the average of the simulated values of 999 FD or PD null models randomly generated, and SD_null_ is the standard deviation of 999 random values [31].

We selected four key traits of waterbirds to calculate the functional diversity: morphological traits (i.e., body mass, body length, bill length, wing length, tarsus length), diet communities (e.g., percentage of invertebrates, fish, seed, fruit, and other plant materials), foraging behavior (e.g., pursuit, pouncing, picking, digging, probing), foraging strata (i.e., below the water surface, on the water surface, on the ground); more details are provided in Supplementary Table S2. The data were sourced from the website https://www.bto.org/ (6 January 2026) and the literature [32,33]. Based on these traits, we calculated Gower distances to convert the species by trait matrix into a functional distance matrix [34]. Subsequently, a functional dendrogram was constructed using hierarchical clustering. Regarding phylogenetic data, we downloaded 10,000 posterior phylogenetic trees from BirdTree (http://birdtree.org, 7 January 2026) using the “Hackett All Species: a set of 10,000 trees with 9993 operational taxonomic units” backbone [35], and matched the species names in our community matrix to the corresponding taxa in the phylogenetic trees using scientific names. All species recorded in our waterbird community were represented in the phylogenetic dataset. We matched the species names in our community matrix to the corresponding taxa in the phylogenetic trees using scientific names. Each phylogenetic tree was pruned to retain only the waterbird species recorded in our study using the “ape” package in R 4.5.2. To account for phylogenetic uncertainty, 999 trees were randomly sampled from the 10,000 posterior trees. A maximum clade credibility (MCC) tree was then generated from these phylogenetic trees [36,37]. Then, abundance-weighted functional and phylogenetic diversity indices were calculated [38].

As functional and phylogenetic diversity metrics are inherently correlated with species richness [39], we hence calculated their standardized effect sizes (SES.FD and SES.PD) to control for this dependence [40]. To generate the expected distributions, the community data matrix was randomized 999 times, with constraints applied to maintain both the observed species richness per community and the regional occurrence frequency of each species [41]. SES.FD and SES.PD were calculated using the R function “ses.pd” in package “picante” [42].

Furthermore, species dominance was determined based on the monthly abundance (P_i_) of each species at the lake scale. Species with Pi > 10% were classified as dominant species [43].

### 2.7. Statistical Analyses

Before modeling, all predictor variables (Supplementary Table S3) were Z-score standardized (mean = 0, standard deviation = 1) to eliminate the influence of differing measurement scales. To mitigate potential multicollinearity among the predictors, we calculated their Variance Inflation Factors (VIFs). Only variables with a VIF value less than 5 were retained in the models [44]. Given that these climatic variables exhibited strong correlations with water level, they were excluded from the final statistical model to avoid multicollinearity. The variables selected after screening are shown in Table 1.

Compared to traditional structural equation models (SEMs), Piecewise structural equation models (SEMs) offer the flexibility to fit multiple generalized or mixed models to assess complex ecological relationships [45,46]. We utilized SEMs to untangle the direct and indirect effects of water level on waterbird diversity. We built SEMs for the annual, wintering (from October to March), and non-wintering periods (from April to September) separately to evaluate temporal dynamics of water level effects. In each SEM, water level was specified as the exogenous driver, environmental variables retained after variable selection were included as mediating variables (Table 1), and three diversity metrics were treated as response variables. To account for pseudo replication, random effects were specified according to the temporal scale: both survey site and month were included as random effects in the annual models while only survey site was included in the wintering and non-wintering models since small sample size may result in unstable variance estimates if months were also included as a random effect. Given the Gaussian distribution of our response variables, Linear Mixed Models (LMMs) were employed to construct the paths within the SEMs. Specifically, at the annual scale, SIDI, NDVI, WL, and WA were included as fixed factors, while month and waterbird observation site were included as random factors. During the wintering and non-wintering periods, WL, WA, and NDVI were included as fixed factors, and waterbird observation site was included as a random effect in the SEMs. Spatial autocorrelation in residuals of liner mixed models was evaluated using the Moran index [47], and no significant spatial autocorrelation was observed (Moran index *p*-values > 0.5). Similarly, temporal autocorrelation was ruled out using the ACF (*p*-values > 0.5) evaluations [48]. Overall model fit was evaluated using tests of directed separation via Fisher’s C statistic [46] with a *p*-value > 0.5 indicating adequate model fit (i.e., no significant missing paths). When the initial model fit was poor (*p* < 0.05), the model was sequentially updated by adding missing paths based on statistical significance and ecological plausibility. To examine whether water level had a direct effect on multidimensional waterbird diversity across different periods, the direct path from water level was removed from the full SEM, and model fit was subsequently assessed using Fisher’s C test [49,50].

Finally, we fitted generalized additive models (GAMs) to further explore the specific response curves (linear or non-linear) of the significant pathways identified by the SEMs. The random structure of models was the same as that specified in the corresponding SEMs. All statistical analyses and data visualizations were performed in R 4.5.2. Specifically, Linear Mixed Models, Piecewise SEMs, and generalized additive models were constructed using the “lme4”, “piecewiseSEM” and “mgcv” packages, respectively [51,52,53]. All graphical plots were generated using the “ggplot2” package.

## 3. Results

### 3.1. The Waterbird Community Composition

In total, 101,110 individuals from 69 waterbird species belonging to seven orders were recorded in our annual survey at Shijiu lake. Based on residency status, these species comprised 34 winter visitors, 11 summer visitors, 18 residents, and 6 passage migrants. Among them, three IUCN globally threatened species were recorded: Oriental Stork (EN), Swan Goose (*Anser cygnoides*, EN), Common Pochard (*Aythya ferina*, VU). One species is listed in the national key protected species Class I: Oriental Stork. Eight species are listed in the national key protected species Class II: Tundra Swan, Swan Goose, Greater White-fronted Goose (*Anser albifrons*), Smew (*Mergellus albellus*), Baikal Teal (*Sibirionetta formosa*), Eurasian Spoonbill, Cotton Pygmy Goose, and Pheasant-tailed Jacana. More details are provided in Supplementary Table S4.

**Figure 3:**
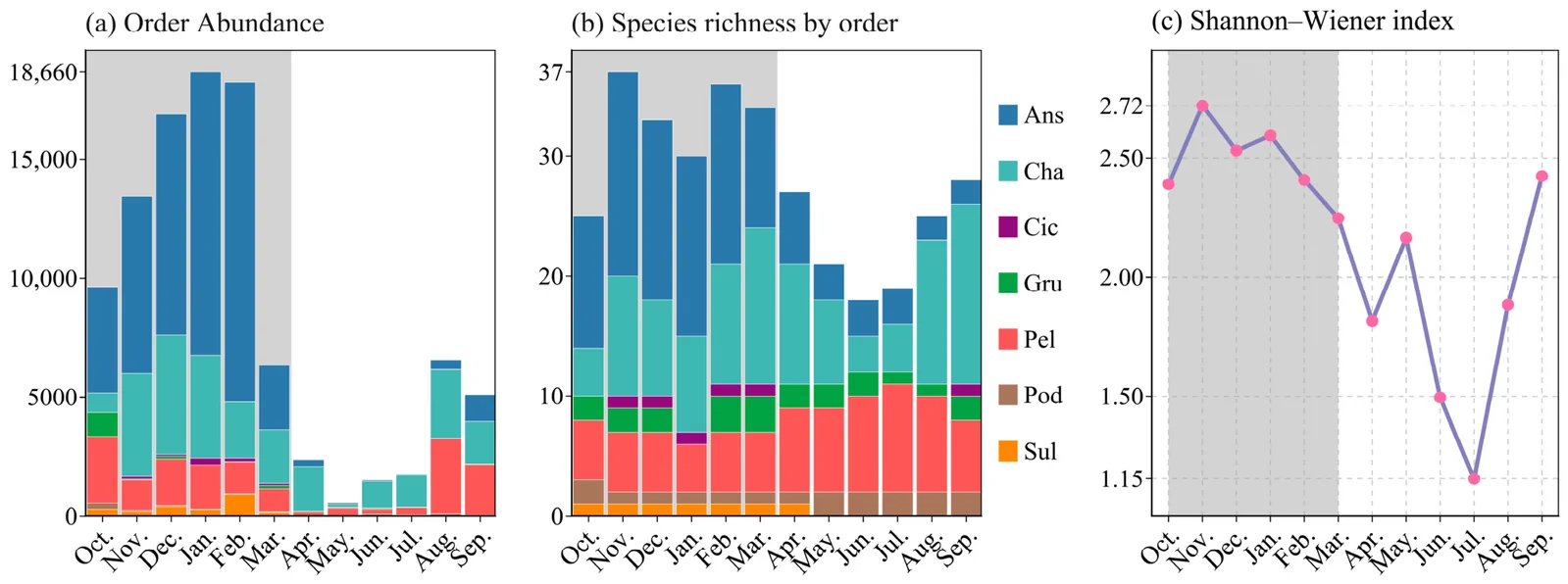
Temporal variations in waterbird community compositions at Shijiu Lake, 2024–2025. (**a**) Monthly abundance of waterbirds by order; (**b**) monthly species richness by order; and (**c**) monthly Shannon–Wiener diversity index of the waterbird community. The gray shaded background indicates the wintering period, while the white background represents the non-wintering period. Abbreviations in the legend denote the following orders: Ans, Anseriformes; Cha, Charadriiformes; Cic, Ciconiiformes; Gru, Gruiformes; Pel, Pelecaniformes; Pod, Podicipediformes; and Sul, Suliformes.

Overall, the waterbird community composition at Shijiu Lake showed pronounced seasonal shifts, with substantial differences between the wintering and non-wintering periods (Figure 3a,b). These shifts were reflected in residency patterns: winter visitors accounted for 87.57–97.62% of the community from November to March, whereas summer visitors became dominant from May to August, reaching a peak of 88.67% in July. Passage migrants were most prevalent in April (70.04%), highlighting the seasonal importance of migration during spring (Supplementary Table S5). Both waterbird abundance and species richness were markedly higher during the wintering period than during the non-wintering period. Similarly, the Shannon–Wiener index was generally higher during the wintering period, peaking in November (2.72), declining to a minimum in July (1.15), and recovering toward autumn (Figure 3c). Meanwhile, the turnover of dominant species was detected, whereas the dominant species during the non-wintering period were relatively uniform (Supplementary Table S6). During winter, the community was dominated by large aggregations of wintering Anseriformes. Following the spring migration, the community underwent a pronounced compositional shift, increasingly dominated by Charadriiformes and Suliformes. Notably, Terns remarkably increased in summer, dominated by breeding Whiskered Tern.

**Figure 4:**
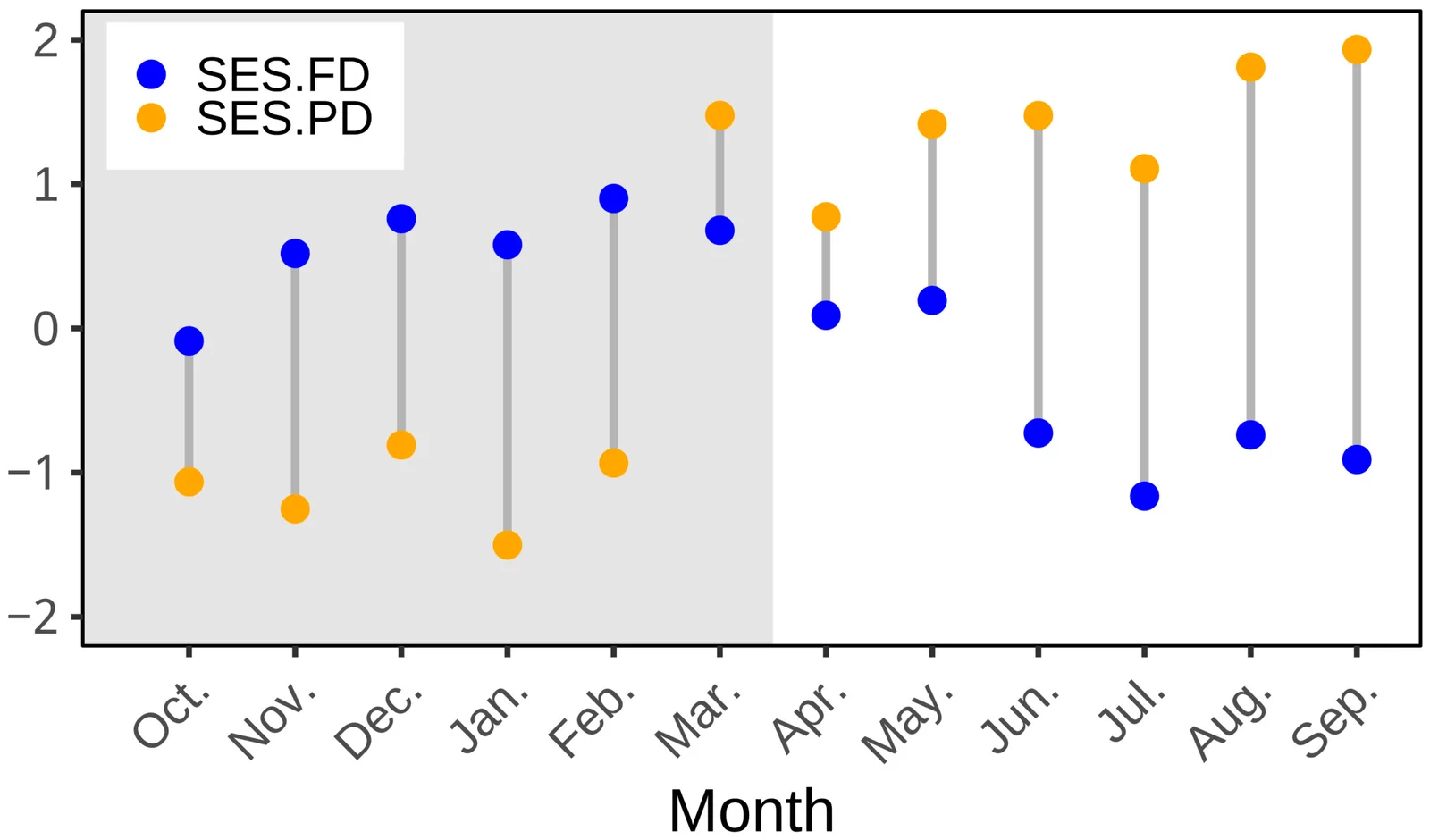
Monthly dynamics of SES.FD and SES.PD for the waterbird community at Shijiu Lake. Blue and orange dots represent the values of SES.FD and SES.PD, respectively. The grey shaded background indicates the wintering period, while the unshaded area represents the non-wintering period.

Meanwhile, both SES.FD and SES.PD showed pronounced seasonal shifts, with substantially opposite patterns between the wintering (shaded) and non-wintering (unshaded) periods. Specifically, SES.FD was generally positive and higher during the wintering period but became negative and lower during the non-wintering period, whereas SES.PD was negative and lower during the wintering period, but became markedly higher during the non-wintering period (Figure 4).

### 3.2. Impact Pathways of Water Level on Waterbird Diversity

**Figure 5:**
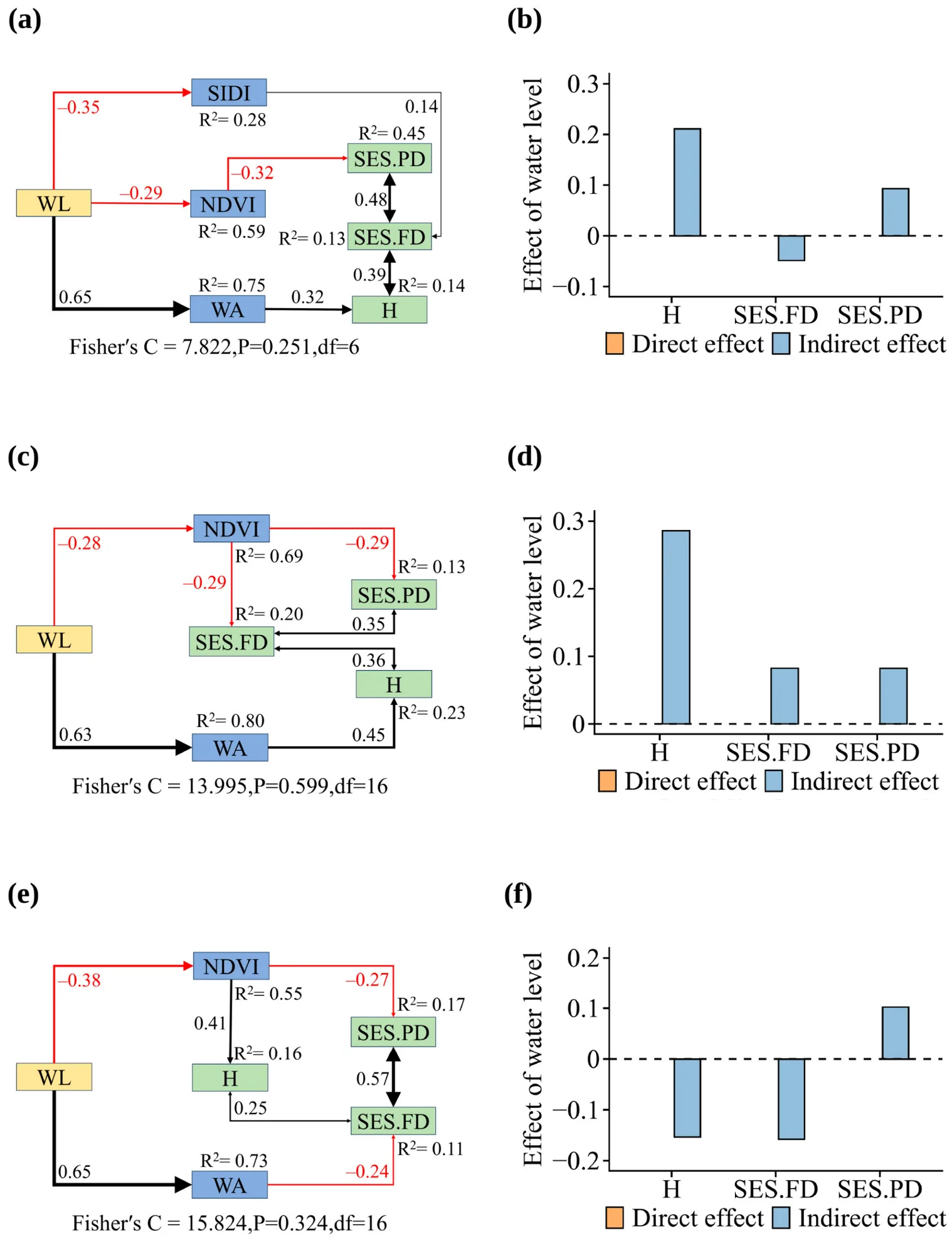
Results of Piecewise structural equation models (SEMs) showing effects of water level on taxonomic, functional, and phylogenetic diversity of waterbirds across temporal scales: (**a**) annual, (**c**) wintering, and (**e**) non-wintering periods. Single-headed arrows represent significant standardized path coefficients (black: positive; red: negative; *p* < 0.05), and double-headed arrows indicate covariances between variables. Panels (**b**,**d**,**f**) present the standardized effects of water level on each diversity dimension in the corresponding periods. Arrow thickness is proportional to the magnitude of the standardized path coefficients. All R^2^ values are marginal determination coefficients. For variable abbreviations see Table 1.

Results of SEMs (Figure 5, all Fisher’s C *p* > 0.05) showed that water level affected multidimensional waterbird diversity exclusively through indirect pathways across all three temporal scales. At the annual scale, higher water level negatively affected the habitat diversity, which therefore influenced waterbird diversities. However, increasing water level not only decreased the NDVI but also increased the size of water area, contributing to a positive indirect effect on the diversity (Figure 5a,b). During the wintering period, increasing water levels expanded the water area and subsequently promoted taxonomic diversity. Higher water level reduced NDVI which also facilitated SES.FD and SES.PD (Figure 5c). Thus, water level exerted positive indirect effects on all diversity dimensions (Figure 5d). Conversely, during the non-wintering period, water area expansion driven by increasing water level negatively affected SES.FD, while higher water level led to a decrease in NDVI, resulting in a negative effect on the taxonomic diversity (Figure 5e). Eventually, water level indirectly suppressed the taxonomic diversity and SES.FD but facilitated SES.FD (Figure 5f).

### 3.3. Effects of Different Variables on Waterbird Diversity

**Figure 6:**
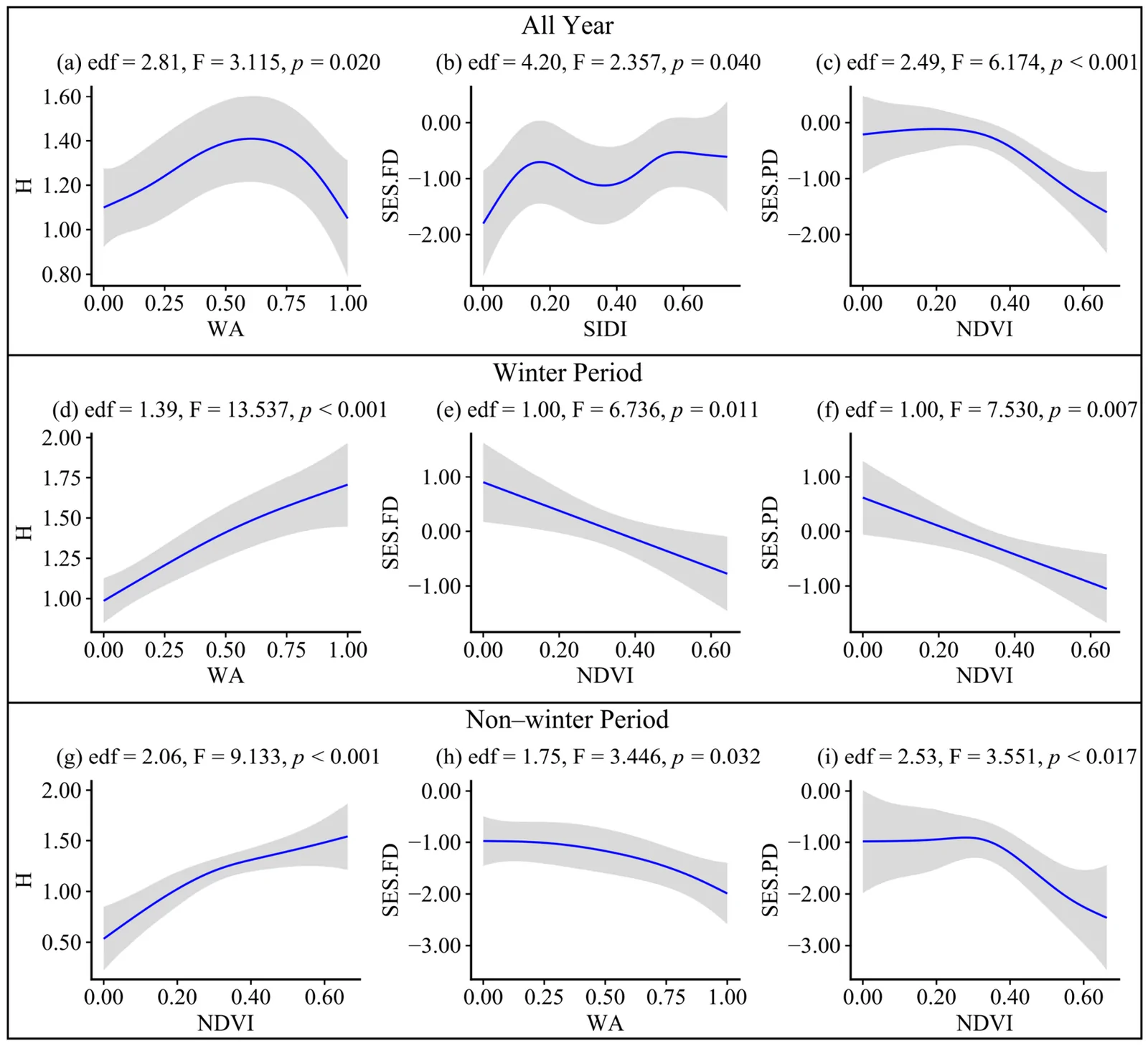
Results of GAMs revealing effects of different variables on waterbird multiple diversities during the annual (**a**–**c**), wintering (**d**–**f**), and non-wintering (**g**–**i**) periods. The vertical axis represents the values of the smoothing function, while the horizontal axis shows the observed values of the explanatory variables. Solid blue lines represent the smooth fitting curve of the waterbird diversity indices and gray shadows indicate the 95% confident interval. For variable abbreviations see Table 1.

Our GAMs revealed complex, temporally dependent responses of waterbird diversity to waterbird habitats (Figure 6). At the annual scale, the taxonomic diversity showed a hump-shaped relationship along with water area (Figure 6a), while SES.FD responded nonlinearly to landscape diversity (SIDI) (Figure 6b), and SES.PD declined with increasing NDVI (Figure 6c). During the wintering period, responses of different diversity indices were mainly monotonic, with the taxonomic diversity increasing almost linearly in response to water area (Figure 6d) and both SES.FD and SES.PD negatively correlated with NDVI (Figure 6e,f). In contrast, during the non-wintering period, the taxonomic diversity initially increased with NDVI and then leveled off (Figure 6g), whereas SES.FD and SES.PD declined with increasing water area (Figure 6h) and NDVI, respectively (Figure 6i).

## 4. Discussion

Hydrodynamics is the priority force in shaping wetland ecosystems. In recessional Yangtze wetlands, seasonal water level fluctuations created excellent habitats both for wintering and non-wintering waterbirds. However, most previous studies focused on wintering periods [54,55,56]. More importantly, the effect pathways of water level on waterbird diversity across different periods is barely explored. Here, using structural equation models, we found that water level fluctuations influence multidimensional waterbird diversity primarily through indirect pathways by reshaping local habitat conditions, rather than through direct effects. In combination with the general additive models, we unveiled that multidimensional waterbird diversities exhibited seasonal specific responses to habitat variables, depending on the different time periods. Accordingly, an adaptive water level regulation strategy that accounts for seasonal variations would be beneficial for improving conservation benefits throughout the year in this region.

### 4.1. Temporal Dynamics of Waterbird Communities

Waterbird communities at Shijiu Lake exhibited clear seasonal dynamics across the annual cycle. Waterbird abundance increased from autumn when the southward migration started and peaked in the wintering period. This result is consistent with studies conducted at Poyang, Dongting and Shengjin lakes in the region [57,58,59]. In addition, waterbird abundance and richness decreased following the wintering period but subsequently increased with the arrival of breeding waterbirds. These results suggest that Shijiu Lake plays an important role in supporting migratory waterbirds throughout different stages of their annual cycle. And the wetland provides habitat for wintering, breeding, and stopover activities and sustains large seasonal assemblages of waterbirds [60]. Interestingly, the functional and phylogenetic diversity of waterbird communities exhibited markedly opposite patterns during the wintering and non-wintering periods (Figure 4). This pattern is strongly associated with fluctuations in waterbird abundance and the seasonal dynamics of dominant species [61,62]. The dominant species during the wintering period are phylogenetically clustered but occupy distinct niches (e.g., *Anser fabalis*, *Mareca falcata*, *Anas crecca*, Supplementary Table S6). In contrast, non-wintering communities are dominated by species with similar ecological niches but distant phylogenetic relationships (e.g., *Chlidonias hybrida*, *Ardea alba*, *Egretta garzetta*).

Meanwhile, water levels dipped in May and did not return to winter levels until June (Figure 2), waterbird abundance had already declined markedly by March, whereas species richness followed a contrasting temporal pattern (Figure 3). This is because most wintering waterbirds (Anseriformes) had migrated northward, leading to a sharp decline in abundance. Concurrently, the influx of passage migrants maintained a relatively stable richness. This community turnover contributed to the contrasting temporal patterns of abundance and order richness [63,64]. The temporal mismatch between the lowest water level and observed changes in waterbird communities suggests the presence of lagged ecological responses, which have been widely documented in wetland ecosystems [65,66]. We recognize this as a limitation of the current study and an important avenue for future investigations. Furthermore, this temporal mismatch should also be considered in the context of the relatively low and prolonged water levels during our study year, amid long-term losses in lake wetlands and hydrological connectivity across the Yangtze River [67]. These conditions underscore the importance of maintaining suitable hydrological regimes and adaptive water level management for conserving multidimensional waterbird diversity.

### 4.2. Pathways of Water Level Affecting Multidimensional Waterbird Diversity

Consistent with our first hypothesis, water level was strongly associated with variation in habitat conditions, particularly with water area. However, its effects on taxonomic, functional, and phylogenetic diversity were primarily indirect and generally modest, operating through habitat mediated pathways identified by the SEMs. Effect of hydrological regimes on waterbird diversity has been widely acknowledged in wetlands space, while pathways are still not clear [68,69]. Water level directly determines the inundation area and duration of wetlands [70,71], which may cascadingly affect habitat quantity and quality for waterbirds [72,73], and ultimately impact waterbird diversity [74,75]. Our SEM models clearly showed that water level affected habitat quantity and quality indirectly across different seasons by shaping habitat characteristic (Figure 5). These results provide fundamental evidence for future conservation practices, calling for more research efforts to unveil the ecological process governing by hydrological regimes.

Our results also supported the second hypothesis that effects of water levels on waterbird diversity varied across seasons. Our result suggested that higher water levels enlarged the size of the open water area, which was subsequently beneficial for waterbird diversity during the wintering period [76] and the whole year round (Figure 6a,d). According to the Island Biogeography theory [77], a larger water area may offer diverse roosting and foraging habitats for waterbirds [78,79] during the wintering period. In addition, a larger area can also reduce the predation risk [80]. Increasing water level also led to a decrease in emergent aquatic vegetation biomass (NDVI as a surrogate), and then jointly affected waterbird diversities (Figure 5).

During the wintering period, an area having sufficient biomass provides abundant food resources for herbivorous and tuber-feeding species, such as geese and swans [81,82]. Moreover, high-NDVI areas also offer shelters for both waterbirds and invertebrates, which are main food for herons and shorebirds [83], attracting more waterbirds [84,85] and contributing to an increase in functional and phylogenetic diversity. During the non-wintering period, increased aquatic vegetation can also provide suitable breeding habitats and food resources for species such as egrets and terns [86,87].

During the non-wintering period, however, higher and more variable water levels may inundate ground nests and destabilize overwater nesting habitats, constraining breeding opportunities for waterbirds [88]. Environmental filtering may further homogenize functional traits within local communities [89], negatively affecting waterbird functional diversity. Following the Habitat Heterogeneity Hypothesis, higher water levels may also decrease the landscape diversity, as some habitat types may be flooded and no longer accessible, harming waterbird diversities [90,91].

## 5. Conclusions

Our study at Shijiu Lake showed that rather than through direct effects, water level fluctuations influenced multidimensional waterbird diversities primarily through indirect pathways by reshaping local habitat conditions. In addition, the direction of these effects varied seasonally. That is, during the wintering period, moderately elevated water levels may be more effective in maintaining higher taxonomic diversity. In contrast, during the non-wintering period, relatively lower water levels may enhance habitat heterogeneity and provide suitable foraging and breeding conditions for diverse waterbird assemblages.

### 5.1. Conservation Implications

These findings underscore the importance of seasonally adaptive water level management for waterbird conservation in wetlands. Following future dam construction at Shijiu Lake, seasonal water level regulation should account for the feasibility of water level manipulation and the need to maintain ecological connectivity. During the wintering period, moderately elevated water levels may help maintain greater water-covered areas and suitable habitats for wintering waterbirds. During the non-wintering period, moderately lower water levels may promote habitat heterogeneity and increase the availability of foraging and breeding habitats, provided that flood-control requirements and necessary water-storage capacity are maintained. Seasonal regulation should be guided by habitat conditions and waterbird responses rather than relying solely on predetermined water level targets. These recommendations may also inform waterbird conservation in other lakes along the Yangtze River where dam construction is planned, with specific measures tailored to local hydrological conditions and conservation objectives.

### 5.2. Limitations

Our study also has some limitations. Since the analysis was conducted based on a single-year dataset, whether the conclusions are generalizable still requires further verification through longer-term data analysis and across multiple wetland sites. Furthermore, given that water level effects may exhibit time lags, we recommend increasing the survey frequency and interpreting multi-source remote sensing images to enable more comprehensive analyses. Finally, as the demand of different guilds varies, future studies may also incorporate waterbird guilds in the analyses to further disentangle the relative importance of local push factors (e.g., habitat deterioration) versus external pull factors (e.g., resource availability in adjacent wetlands).

## Figures and Tables

**Table 1:** Variables (with their abbreviations) retained in our final models.

Variables	Definitions
WL (m)	Lake water levels matched to remote sensing acquisition data.
WA (%)	Proportion of open water area within a 1.5 km radius of each observation site at Shijiu lake.
NDVI	Vegetation condition within a 1.5 km radius of each observation site at Shijiu lake.
SIDI	Landscape diversity and dominance patterns within a 1.5 km radius of each observation site at Shijiu lake.

## Data Availability

The raw data supporting the conclusions of this article will be made available by the authors upon request.

## Supplementary Materials

The following supporting information can be downloaded at https://www.mdpi.com/article/10.3390/ani16213354/s1, Supplementary Table S1: The remote sensing image used in the study and the corresponding water level on the day of the image (the reference level zero: Wusong Elevation); Supplementary Table S2: Functional traits of waterbirds; Supplementary Table S3: Environmental variables and corresponding definitions in this study; Supplementary Table S4: Waterbird species included in this study (resident, R; winter birds, W; summer visitor, S; passage migrant, P; * breeding birds); Supplementary Table S5: Monthly proportions of waterbirds belonging to different residency types; Supplementary Table S6: Dominant waterbird species in Shijiu Lake across different months; Supplementary Table S7: Fisher´s C tests of structural equation models evaluating the direct effects of water level on multidimensional waterbird diversity across different periods (“…” indicates random effect); Supplementary Table S8: The results for WA, NDVI, and SIDI at each sampling site in different months are arranged in counterclockwise order, starting with the site closest to Sheshan Sluice; Supplementary Figure S1: Daily water level dynamics from 1 October 2014 to 31 October 2024.

## Abbreviations

The following abbreviations are used in this manuscript:

WL	Lake water levels matched to remote sensing acquisition dates
WA	Proportion of open water area within a 1.5 km radius of each observation site at Shijiu lake
NDVI	Vegetation condition within a 1.5 km radius of each observation site at Shijiu lake
SIDI	Landscape diversity and dominance patterns within a 1.5 km radius of each observation site at Shijiu lake
H	Shannon–Wiener index
SES.PD	The standardized effect value of the functional diversity index
SES.FD	The standardized effect value of the phylogenetic diversity index
