# Supplementary material for: Impacts of Water Level on Multidimensional Waterbird Diversity in a Wetland of Yangtze River Floodplain: Insights for Conservation
Source: Animals (Basel). 2026 Oct 24;16(21):3354. doi: 10.3390/ani16213354 (PMC13762051; doi:10.3390/ani16213354)
Supplement: Supplementary file 1 [file animals-16-03354-s001.zip › animals-4595422-supplementary.pdf]

Supplementary Table S1. The remote sensing image used in the study and the corresponding water level on the day of the image (the reference level zero: Wusong Elevation).

NO.	Image date	Water level on image data (m)	Satellite
1	2024/8/9	9.73	Landsat-8
2	2024/11/29	5.03	Landsat-8
3	2024/12/31	4.90	Landsat-8
4	2025/1/8	4.99	Landsat-8
5	2025/2/9	5.00	Landsat-8
6	2025/3/21	4.92	Landsat-8
7	2025/4/14	4.61	Landsat-8
8	2025/5/25	4.99	Sentinel-2
9	2025/6/17	7.58	Landsat-8
10	2025/7/3	8.55	Landsat-8
11	2025/8/28	5.57	Landsat-8
12	2025/9/22	5.95	Sentinel-2
13	2025/10/29	7.23	Sentinel-2

Supplementary Table S2. Functional traits of waterbirds.

Functional Category	Variable Type	Traits	Description
Morphological Traits	Continuous	Body mass	Total body mass of the bird (in grams)
		Body length	Length of the bird from the tip of the bill to the end of the tail (in millimeters)
		Bill length	Length of the bill from tip to base (in millimeters)
		Wing length	Length of a single wing when folded (in millimeters)
		Tarsus length	Length of the tarsus bone (in millimeters)
Dite Type	Percentage variable (0–100%)	Diet Inv	Invertebrates-general, aquatic invertebrates, shrimp, krill, squid, worms, insect, et al.
		Diet Vend	Mammals, Birds
		Diet Vect	Reptiles, snakes, amphibians, salamanders
		Diet Vfish	Fish
		Diet Vunk	Vertebrates-general or unknown
		Diet Scav	Scavenge, garbage, offal, carcasses, trawlers, carrrion
		Diet Fruit	Fruits and berries
		Diet Seed	Seed, maize, nuts, spores, wheat, grains
		Diet Plant	Grass, tubers, leaves, above ground vegetation, shoots, aquatic vegetation, et al.
Foraging Method	Binary Variable	Pursuit	Actively chasing prey
		Pouncing	Ambushing prey from a concealed position
		Picking	Picking food items from surfaces
		Grazing	Grazing on vegetation or small organisms
		Digging	Digging into substrates to extract food
		Scavenging	Sweeping the bill through water to capture prey
		Probing	Probing into soft substrates to locate food
Foraging Habitat	Percentage variable (0–100%)	ForStrat1	Foraging below the water surfaces
		ForStrat2	Foraging on or just (<5 inches) below water surface
		ForStrat3	Foraging on ground

Supplementary Table S3. Environmental variables and corresponding definitions in this study.

Variables	Definitions
WL (m)	Lake water levels matched to remote sensing acquisition dates
WL_CV	The monthly fluctuation in water levels of the Shijiu lake
Temp_M(℃)	Daily average temperature
Temp_CV	Monthly temperature fluctuation
Precip (mm)	Daily precipitation
Precip_CV	Monthly precipitation fluctuation
WA (%)	Area proportion of open water area within a 1.5 km radius of each observation site in Shijiu lake
GA (%)	Area proportion of Grass area within a 1.5 km radius of each observation site in Shijiu lake
MA (%)	Area proportion of Mudflat area within a 1.5 km radius of each observation site in Shijiu lake
VA (%)	Area proportion of emergent vegetation area within a 1.5 km radius of each observation site in Shijiu lake
NDVI	Vegetation condition within a 1.5 km radius of each observation site in Shijiu lake
NDVI_CV	Spatial variability of vegetation condition within a 1.5 km radius of each observation site in Shijiu lake
MNDWI	Extent of open water within a 1.5 km radius of each observation site in Shijiu lake
MNDWI_CV	Spatial variability of water condition within a 1.5 km radius of each observation site in Shijiu lake
SIDI	Landscape diversity and dominance patterns within a 1.5 km radius of each observation site in Shijiu lake
SIEI	Landscape evenness within a 1.5 km radius of each observation site in Shijiu lake

Supplementary Table S4. Waterbird species included in this study (resident, R; winter birds, W; summer visitor, S; passage migrant, P; * breeding birds).

Order	Family	Common name	Scientific name	IUCN	Resident
ANSERIFORMES	Anatidae	Tundra Swan	*Cygnus columbianus*	LC	W
		Greylag Goose	*Anser anser*	LC	W
		Swan Goose	*Anser cygnoides*	EN	W
		Taiga Bean Goose	*Anser fabalis*	LC	W
		Greater White-fronted Goose	*Anser albifrons*	LC	W
		Smew	*Mergellus albellus*	LC	W
		Common Merganser	*Mergus merganser*	LC	W
		Common Shelduck	*Tadorna tadorna*	LC	W
		Ruddy Shelduck	*Tadorna ferruginea*	LC	W
		Cotton Pygmy Goose	*Nettapus coromandelianus*	LC	S*
		Common Pochard	*Aythya ferina*	VU	W
		Tufted Duck	*Aythya fuligula*	LC	W
		Garganey	*Spatula querquedula*	LC	W
		Northern Shoveler	*Spatula clypeata*	LC	W
		Baikal Teal	*Sibirionetta formosa*	LC	W
		Falcated Duck	*Mareca falcata*	LC	W
		Gadwall	*Mareca strepera*	LC	W
		Eurasian Wigeon	*Mareca penelope*	LC	W
		Chinese Spot-billed Duck	*Anas zonorhyncha*	LC	W
		Mallard	*Anas platyrhynchos*	LC	W
		Northern Pintail	*Anas acuta*	LC	W
		Eurasian Teal	*Anas crecca*	LC	W
PODICIPEDIFORMES	Podicipedidae	Little Grebe	*Tachybaptus ruficollis*	LC	R*
		Great Crested Grebe	*Podiceps cristatus*	LC	R*
GRUIFORMES	Rallidae	Brown-cheeked Rail	*Rallus indicus*	LC	P
		Common Moorhen	*Gallinula chloropus*	LC	R*
		Eurasian Coot	*Fulica atra*	LC	W
CICONIIFORMES	Ciconiidae	Oriental Stork	*Ciconia boyciana*	EN	W
PELECANIFORMES	Threskiornithidae	Eurasian Spoonbill	*Platalea leucorodia*	LC	W
	Ardeidae	Yellow Bittern	*Ixobrychus sinensis*	LC	S*
		Black-crowned Night Heron	*Nycticorax nycticorax*	LC	R*
		Striated Heron	*Butorides striata*	LC	S*
		Chinese Pond Heron	*Ardeola bacchus*	LC	S*
		Eastern Cattle Egret	*Bubulcus coromandus*	LC	S*
		Grey Heron	*Ardea cinerea*	LC	W
		Purple Heron	*Ardea purpurea*	LC	S*
		Great Egret	*Ardea alba*	LC	W
		Intermediate Egret	*Ardea intermedia*	LC	S*

Order	Family	Common name	Scientific name	IUCN	Resident
		Little Egret	*Egretta garzetta*	LC	R*
SULIFORMES	Phalacrocoracidae	Great Cormorant	*Phalacrocorax carbo*	LC	W
CHARADRIIFORMES	Recurvirostridae	Pied Avocet	*Recurvirostra avosetta*	LC	W
		Black-winged Stilt	*Himantopus himantopus*	LC	P
	Charadriidae	Northern Lapwing	*Vanellus vanellus*	NT	W
		Grey-headed Lapwing	*Vanellus cinereus*	LC	S*
		Pacific Golden Plover	*Pluvialis fulva*	LC	P
		Long-billed Plover	*Charadrius placidus*	LC	P
		Little Ringed Plover	*Charadrius dubius*	LC	P
		Kentish Plover	*Charadrius alexandrinus*	LC	W
	Rostratulidae	Greater Painted snipe	*Rostratula benghalensis*	LC	R*
	Jacanidae	Pheasant-tailed Jacana	*Hydrophasianus chirurgus*	LC	S*
	Scolopacidae	Eurasian Whimbrel	*Numenius phaeopus*	LC	P
		Bar-tailed Godwit	*Limosa lapponica*	NT	P
		Black-tailed Godwit	*Limosa limosa*	NT	P
		Ruff	*Calidris pugnax*	LC	P
		Red-necked Stint	*Calidris ruficollis*	NT	P
		Dunlin	*Calidris alpina*	NT	W
		Common Snipe	*Gallinago gallinago*	LC	P
		Common Sandpiper	*Actitis hypoleucos*	LC	P
		Green Sandpiper	*Tringa ochropus*	LC	W
		Spotted Redshank	*Tringa erythropus*	LC	P
		Common Greenshank	*Tringa nebularia*	LC	P
		Common Redshank	*Tringa totanus*	LC	P
		Wood Sandpiper	*Tringa glareola*	LC	P
		Marsh Sandpiper	*Tringa stagnatilis*	LC	P
	Glareolidae	Oriental Pratincole	*Glareola maldivarum*	LC	S
	Laridae	Black-headed Gull	*Chroicocephalus ridibundus*	LC	W
		Vega Gull	*Larus vegae*	LC	W
		Common Tern	*Sterna hirundo*	LC	P
		Whiskered Tern	*Chlidonias hybrida*	LC	S*

Supplementary Table S5. Monthly proportions of waterbirds belonging to different residency types.

Month	Resident (R) %	Winter visitor (W)%	Summer visitor (S)%	Passage migrant (P)%
November	0.51%	90.03%	1.92%	7.53%
December	0.40%	96.24%	0.18%	3.18%
January	0.11%	96.74%	0.06%	3.09%
February	0.29%	97.62%	0.44%	1.66%
March	2.90%	87.57%	3.22%	6.31%
April	2.66%	21.18%	6.12%	70.04%
May	13.62%	21.86%	56.63%	7.89%
June	9.51%	6.72%	83.77%	0.00%
July	5.05%	6.17%	88.67%	0.11%
August	2.22%	27.33%	67.58%	2.86%
September	1.06%	60.04%	33.93%	4.98%
October	4.53%	86.47%	0.47%	8.53%

Supplementary Table.S6 Dominant waterbird species in Shijiu Lake across different months.

Month	Region	Dominant species	Abundance	Proportion
November	Lake	*Calidris alpina*	2465	18.34%
		Sibirionetta formosa	2460	18.31%
		Anser fabalis	1975	14.70%
December	Lake	*Anser fabalis*	4265	25.26%
		Anas crecca	2292	13.58%
		Calidris alpina	1920	11.37%
January	Lake	*Anser fabalis*	4012	21.50%
		Anas crecca	2700	14.47%
		Mareca falcata	2004	10.74%
February	Lake	*Anser fabalis*	6114	33.54%
		Mareca falcata	2100	11.52%
March	Lake	*Anas crecca*	1969	31.05%
		Recurvirostra avosetta	1755	27.68%
April	Lake	*Tringa erythropus*	1401	59.11%
May	Lake	*Egretta garzetta*	230	41.22%
June	Lake	*Chlidonias hybrida*	1098	73.05%
July	Lake	*Chlidonias hybrida*	1360	76.28%
August	Lake	*Chlidonias hybrida*	2523	38.40%
		Ardea alba	1303	19.83%
		Egretta garzetta	1248	18.99%
September	Lake	*Ardea alba*	997	19.54%
		Chlidonias hybrida	890	17.44%
		Anas zonorhyncha	569	11.15%
		Anas platyrhynchos	550	10.78%
October	Lake	*Platalea leucorodia*	2088	21.68%
		Anas zonorhyncha	2015	20.92%

Supplementary Table.S7 Fisher´s C tests of structural equation models evaluating the direct effects of water level on multidimensional waterbird diversity across different periods ("…"indicates random effect).

Period	Model	Fisher´s C	df	P
Full year	H ~ WA + NDVI + SIDI + …	11.290	8	0.186
	SES.FD ~ WA + NDVI + SIDI + …	9.971	8	0.267
	SES.FD ~ WA + NDVI + SIDI + …	8.369	8	0.398
Wintering	H ~ WA + NDVI + …	5.328	4	0.225
	SES.FD ~ WA + NDVI + …	5.761	4	0.218
	SES.PD ~ WA + NDVI + …	3.956	4	0.412
Non-wintering	H ~ WA + NDVI + …	6.91	4	0.141
	SES.FD ~ WA + NDVI + …	4.197	4	0.380
	SES.PD ~ WA + NDVI + …	5.882	4	0.208

Supplementary Figure S1. Daily water level dynamics from 1 October 2014 to 31 October 2024.

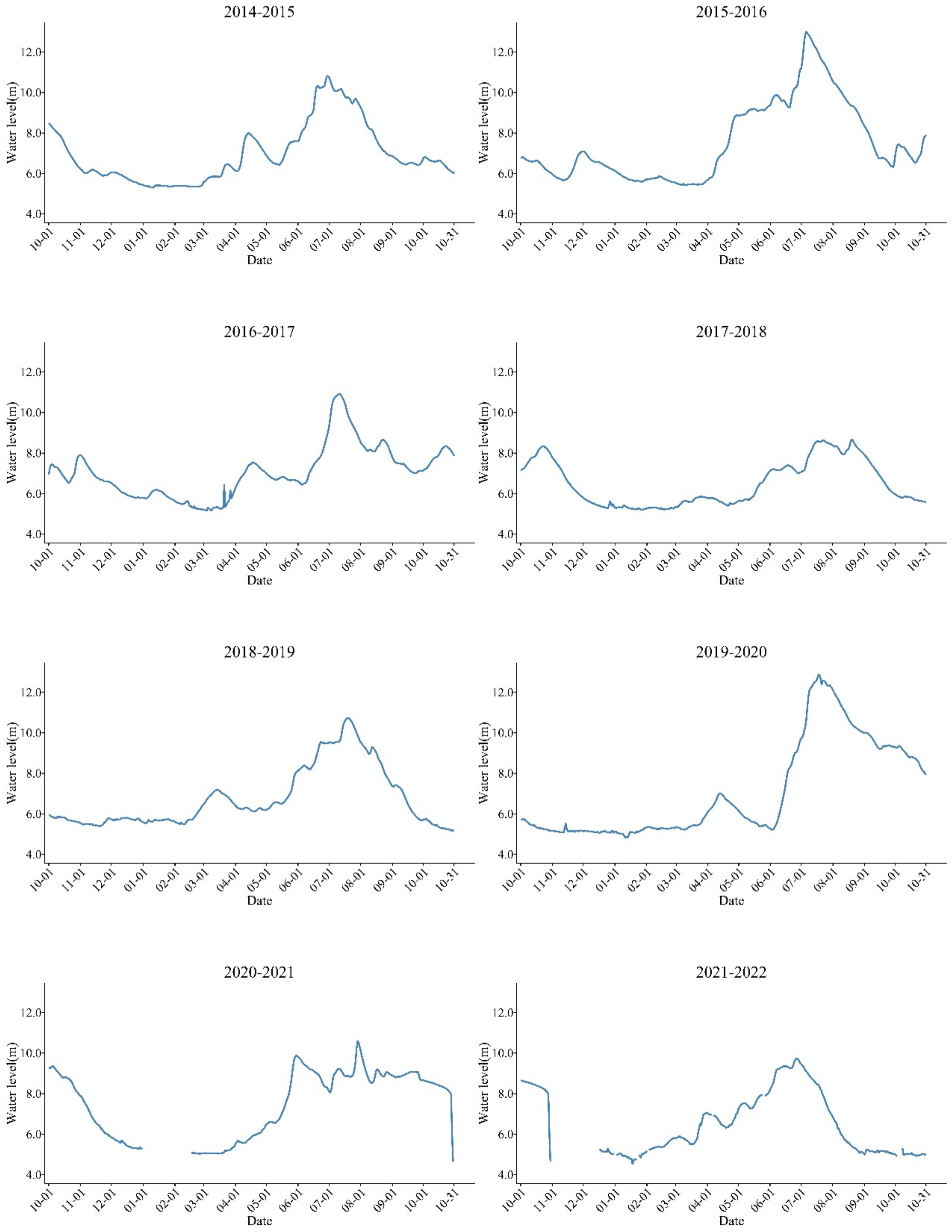

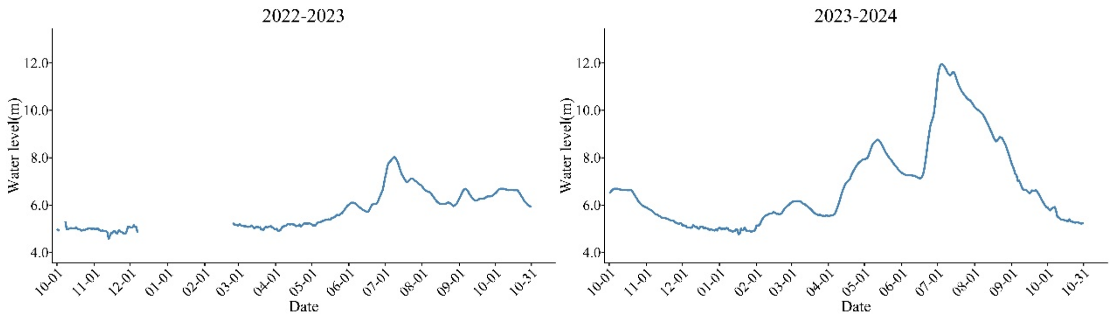

2022-2023
2023-2024
Water level(m)
12.0
10.0
8.0
6.0
4.0
10-01
11-01
12-01
01-01
02-01
03-01
04-01
05-01
06-01
07-01
08-01
09-01
10-01
10-31
Date

Supplementary Table.S8 The results for WA, NDVI, and SIDI at each sampling site in different months are arranged in counterclockwise order, starting with the site closest to Sheshan Sluice.

Month	WA	NDVI	SIDI
Oct.	0.9466	0.2865	0.1026
Oct.	0.9195	0.2840	0.1508
Oct.	0.9433	0.2821	0.1091
Oct.	0.8827	0.2325	0.2168
Oct.	0.8430	0.3117	0.2772
Oct.	0.9696	0.2187	0.0595
Oct.	0.9788	0.2879	0.0416
Oct.	1.0000	0.0000	0.0000
Oct.	0.9841	0.2004	0.0278
Oct.	0.9802	0.2234	0.4856
Oct.	0.4276	0.2694	0.6542
Oct.	0.7500	0.2769	0.3920
Oct.	0.1606	0.2227	0.7309
Oct.	0.1263	0.2640	0.6820
Oct.	0.1238	0.3175	0.5696
Oct.	0.8892	0.2500	0.2027
Nov.	0.4989	0.2986	0.5648
Nov.	0.3161	0.3087	0.6314
Nov.	0.6674	0.3250	0.4936
Nov.	0.4980	0.3645	0.6069
Nov.	0.4281	0.3840	0.6409
Nov.	0.4202	0.2758	0.5629
Nov.	0.5113	0.2491	0.5230
Nov.	0.9889	0.0000	0.3504
Nov.	0.0030	0.2472	0.0800
Nov.	0.0374	0.3591	0.6066
Nov.	0.1653	0.6039	0.7041
Nov.	0.0546	0.5000	0.6373
Nov.	0.0492	0.3649	0.5805
Nov.	0.0141	0.5572	0.6221
Nov.	0.0339	0.6202	0.5181
Nov.	0.0413	0.4900	0.5285
Dec.	0.4260	0.2296	0.5793
Dec.	0.2436	0.2442	0.5131
Dec.	0.5214	0.2857	0.5606
Dec.	0.1710	0.2645	0.4880
Dec.	0.2947	0.2781	0.6323
Dec.	0.0212	0.2138	0.1283
Dec.	0.4143	0.1890	0.5044
Dec.	0.8079	0.0000	0.5276
Dec.	0.0079	0.2022	0.0972

Month	WA	NDVI	SIDI
Dec.	0.0271	0.2813	0.4729
Dec.	0.0212	0.4316	0.6033
Dec.	0.0159	0.3899	0.3873
Dec.	0.0208	0.2779	0.6095
Dec.	0.0048	0.3800	0.5886
Dec.	0.0154	0.4151	0.4991
Dec.	0.0404	0.3919	0.4714
Jan.	0.4414	0.3034	0.5340
Jan.	0.2071	0.3254	0.4265
Jan.	0.4733	0.3674	0.5459
Jan.	0.0881	0.3530	0.3382
Jan.	0.2750	0.3391	0.5875
Jan.	0.0226	0.2829	0.1093
Jan.	0.4150	0.2499	0.4952
Jan.	0.7752	0.0000	0.5514
Jan.	0.0036	0.2673	0.0395
Jan.	0.0288	0.3811	0.5977
Jan.	0.0677	0.5599	0.6633
Jan.	0.0119	0.5213	0.4592
Jan.	0.0345	0.4028	0.5072
Jan.	0.0025	0.5394	0.6234
Jan.	0.0216	0.5855	0.5114
Jan.	0.0482	0.5280	0.5152
Feb.	0.4638	0.2812	0.5701
Feb.	0.2955	0.2934	0.5300
Feb.	0.7181	0.3114	0.4317
Feb.	0.0067	0.2741	0.2238
Feb.	0.1103	0.2892	0.4521
Feb.	0.0150	0.2448	0.2815
Feb.	0.2962	0.2362	0.6450
Feb.	0.5892	0.0000	0.6351
Feb.	0.0041	0.2633	0.4089
Feb.	0.0293	0.3044	0.6533
Feb.	0.0180	0.3478	0.6334
Feb.	0.0115	0.3935	0.3661
Feb.	0.0239	0.3108	0.5209
Feb.	0.0161	0.3427	0.5716
Feb.	0.0185	0.3466	0.5064
Feb.	0.0403	0.4211	0.4225
Mar.	0.3830	0.4041	0.6916
Mar.	0.0514	0.4053	0.5323
Mar.	0.2318	0.4271	0.6242
Mar.	0.0558	0.3726	0.5168

Month	WA	NDVI	SIDI
Mar.	0.1995	0.3887	0.6921
Mar.	0.0053	0.3890	0.5013
Mar.	0.2367	0.3678	0.5694
Mar.	0.7470	0.0000	0.5694
Mar.	0.0043	0.4396	0.6619
Mar.	0.0171	0.4786	0.5526
Mar.	0.0361	0.5091	0.1421
Mar.	0.0000	0.5949	0.0934
Mar.	0.0030	0.4682	0.6086
Mar.	0.0000	0.5778	0.5114
Mar.	0.0241	0.5721	0.4854
Mar.	0.0567	0.6426	0.1866
Apr.	0.4579	0.4184	0.6644
Apr.	0.1039	0.4268	0.5866
Apr.	0.0869	0.4460	0.4071
Apr.	0.0389	0.3913	0.4560
Apr.	0.0531	0.4270	0.5760
Apr.	0.0232	0.3652	0.5344
Apr.	0.0910	0.3850	0.4436
Apr.	0.7593	0.0000	0.5621
Apr.	0.0017	0.4523	0.4604
Apr.	0.0376	0.4963	0.5267
Apr.	0.1340	0.5944	0.2974
Apr.	0.0259	0.6549	0.1310
Apr.	0.0156	0.5510	0.5713
Apr.	0.0208	0.6497	0.5210
Apr.	0.0600	0.6507	0.4895
Apr.	0.0507	0.6629	0.3081
May.	0.1567	0.2581	0.6606
May.	0.0629	0.2773	0.5840
May.	0.2246	0.2465	0.5441
May.	0.2116	0.2513	0.6926
May.	0.3430	0.2575	0.7061
May.	0.1321	0.2979	0.6097
May.	0.2540	0.3052	0.6643
May.	0.8797	0.3087	0.4682
May.	0.0528	0.2889	0.3316
May.	0.2926	0.2566	0.7235
May.	0.1748	0.2906	0.5051
May.	0.0041	0.3395	0.0773
May.	0.0000	0.3983	0.4701
May.	0.0172	0.3109	0.5178
May.	0.0262	0.3085	0.4610

Month	WA	NDVI	SIDI
May.	0.0537	0.2940	0.4203
Jun.	0.7974	0.2927	0.3453
Jun.	0.7716	0.2771	0.3793
Jun.	0.7828	0.2616	0.3569
Jun.	0.8368	0.3257	0.2813
Jun.	0.8727	0.2767	0.2290
Jun.	0.9647	0.2096	0.4933
Jun.	0.9925	0.3013	0.0159
Jun.	1.0000	0.0000	0.0000
Jun.	0.9914	0.2071	0.4065
Jun.	1.0000	0.0000	0.0000
Jun.	0.8296	0.2786	0.3012
Jun.	0.6742	0.3134	0.5102
Jun.	0.0385	0.3935	0.5137
Jun.	0.5785	0.3316	0.5787
Jun.	0.8934	0.3161	0.1931
Jun.	0.9356	0.2598	0.1250
Jul.	0.6641	0.3569	0.5099
Jul.	0.5565	0.3617	0.5797
Jul.	0.7480	0.3222	0.4030
Jul.	0.6781	0.3335	0.4745
Jul.	0.7240	0.3738	0.4570
Jul.	0.4565	0.2273	0.5199
Jul.	0.8574	0.2923	0.2570
Jul.	1.0000	0.0000	0.0000
Jul.	0.8905	0.2626	0.2054
Jul.	0.9847	0.0000	0.4892
Jul.	0.3574	0.3041	0.6751
Jul.	0.5550	0.3501	0.5664
Jul.	0.0246	0.5348	0.3438
Jul.	0.3657	0.3869	0.6498
Jul.	0.6292	0.3990	0.5411
Jul.	0.9020	0.3079	0.1799
Aug.	0.5852	0.3420	0.5908
Aug.	0.3809	0.3490	0.6744
Aug.	0.7253	0.3939	0.4458
Aug.	0.3818	0.3847	0.7009
Aug.	0.5928	0.4004	0.5851
Aug.	0.4936	0.3226	0.6258
Aug.	0.8416	0.3859	0.2751
Aug.	1.0000	0.0000	0.0000
Aug.	0.5010	0.2928	0.5892
Aug.	0.6623	0.3142	0.6465

Month	WA	NDVI	SIDI
Aug.	0.1786	0.5407	0.6268
Aug.	0.0488	0.3683	0.2158
Aug.	0.0000	0.4778	0.4647
Aug.	0.0000	0.4666	0.4954
Aug.	0.0184	0.5283	0.4366
Aug.	0.0181	0.3291	0.5098
Sep.	0.7836	0.4241	0.3597
Sep.	0.7276	0.4016	0.4401
Sep.	0.8383	0.4673	0.2860
Sep.	0.5904	0.3862	0.5883
Sep.	0.5580	0.4053	0.6170
Sep.	0.7281	0.4011	0.4339
Sep.	0.9621	0.3462	0.0734
Sep.	1.0000	0.0000	0.0000
Sep.	0.8430	0.3714	0.2747
Sep.	0.9737	0.3953	0.4925
Sep.	0.3140	0.5679	0.6528
Sep.	0.0884	0.5029	0.2445
Sep.	0.0270	0.4864	0.4759
Sep.	0.0058	0.5443	0.5058
Sep.	0.0509	0.5497	0.4676
Sep.	0.4031	0.4920	0.6105
